# Temporal Trends in Vertebral Size and Shape from Medieval to Modern-Day

**DOI:** 10.1371/journal.pone.0004836

**Published:** 2009-03-12

**Authors:** Juho-Antti Junno, Markku Niskanen, Miika T. Nieminen, Heli Maijanen, Jaakko Niinimäki, Risto Bloigu, Juha Tuukkanen

**Affiliations:** 1 Department of Anatomy and Cell Biology, University of Oulu, Oulu, Finland; 2 Department of Arts and Anthropology, University of Oulu, Oulu, Finland; 3 Department of Diagnostic Radiology, Oulu University Hospital, Oulu, Finland; 4 Medical Informatics Group, University of Oulu, Oulu, Finland; University of Utah, United States of America

## Abstract

Human lumbar vertebrae support the weight of the upper body. Loads lifted and carried by the upper extremities cause significant loading stress to the vertebral bodies. It is well established that trauma-induced vertebral fractures are common especially among elderly people. The aim of this study was to investigate the morphological factors that could have affected the prevalence of trauma-related vertebral fractures from medieval times to the present day. To determine if morphological differences existed in the size and shape of the vertebral body between medieval times and the present day, the vertebral body size and shape was measured from the 4th lumbar vertebra using magnetic resonance imaging (MRI) and standard osteometric calipers. The modern samples consisted of modern Finns and the medieval samples were from archaeological collections in Sweden and Britain. The results show that the shape and size of the 4th lumbar vertebra has changed significantly from medieval times in a way that markedly affects the biomechanical characteristics of the lumbar vertebral column. These changes may have influenced the incidence of trauma- induced spinal fractures in modern populations.

## Introduction

Osteoporosis-related fractures are an increasingly common problem for developed world healthcare. Historical and archeological evidence for osteoporosis has been investigated in several studies and bone fragility has been examined through several aspects such as bone geometry, trabecular structure and bone mineral density [1], [2], [3]. Previous studies of bone structure and density in the proximal femur have demonstrated that temporal trends have produced phenotypes with varied characteristics in bone biomechanics [4] and for this study it was assumed that similar trends might be seen in vertebrae.

Low energy vertebral crush fractures are the most typical clinical expression of osteoporosis. Reduction in vertebral height by 20% is considered to indicate spine fracture [5] and this value is also used for assessing osteoporosis from archaeological vertebrae. Low energy spine fractures are common in both elderly men and women, postmenopausal women being the group that is most at risk [6]–[8].

There are several mechanisms that contribute to the appearance of vertebral fractures. Bone mineral density (BMD) and bone mineral content are traditionally linked to the pathogenesis of bone fragility [9]. According to Biggeman et al. [10] there is a strong correlation (R = 0.91) between vertebral strength and product of vertebral bone density and endplate area. The role of reduced vertebral cross sectional area (CSA) as a risk factor for vertebral fractures is also widely recognized. Men and women with vertebral fractures have similar vertebral height relative to controls but often reduced vertebral width[11]–[13]. Ruyssen-Witrand and co-workers have proposed that “vertebral size should be considered as a potential independent vertebral fracture risk factor” [14].

Vertebral size is controlled by both genetic and developmental factors. Factors that result in reduced bone size during growth include malnutrition and low levels of physical activity. Vertebral bodies are already 17% larger in boys than in girls by Tanner growth stage I, bone densities being similar in both sexes and the difference continues to increase being greatest at stage V. [15] Subperiosteal bone formation increases the vertebral width in men resulting in a decreased fracture risk [16].

The mechanism for larger vertebral CSA in males is probably connected to higher level of physical activity [17], [18] and greater muscle mass in boys [15], with vertebral CSA being phenotypically pliant. Increased vertebral CSA is connected, except to normal periosteal growth, during adulthood also possibly to resorption and remodeling in vertebral body [19].

Sex differences in vertebral strength resulting in more frequent spine fractures in women cannot be explained by sex differences in BMD or trabecular bone volume [20], [21], but there are differences in vertebral size [13]. However this finding is controversial as sexual distinction in vertebral cross-sectional area is partially explained by the larger stature and overall body size in men. In the end there is no significant difference between men and women in vertebral load per cross sectional area unit [16], [22], [23].

The assumption that reduced cross-sectional area (computed from anteroposterior and mediolateral dimensions) of lumbar vertebrae will predispose to spine fractures, was the basis for this study. The vertebral characteristics of medieval and modern people were compared with the aim of discovering potential temporal differences in vertebral morphology. The object for measurements was the fourth lumbar vertebra (L4). Vertebral fractures are usually more common in the lumbar and lower thoracic spine. The reason for selecting L4 was the heavy loads supported by this vertebra and its good preservation in archeological material. L5, in theory, is even more prone for loading. L5 was excluded as variations in vertebral morphology are frequent in L5, especially in the region of the posterior corpus.

## Results

**Figure 1:**
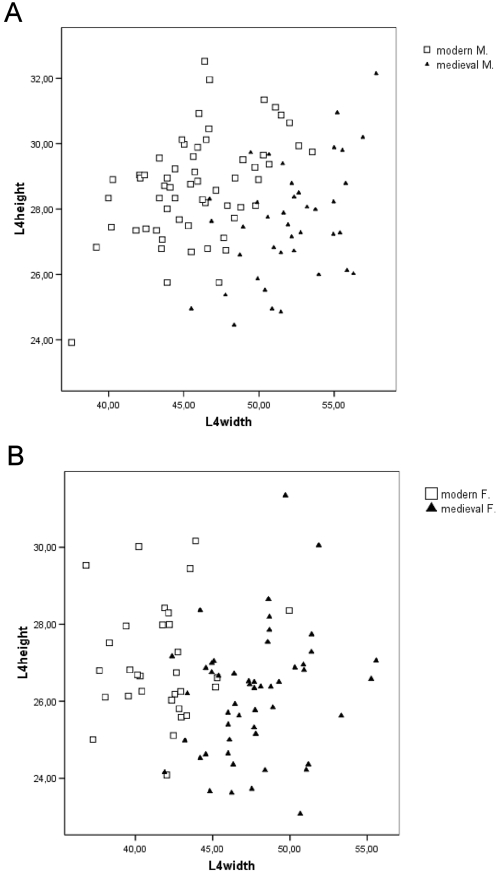
The connection between vertebral height and width (in mm). A: male samples B: female samples.

According to the analysis represented here, clear temporal trend from medieval to modern day was found in dimensions of fourth lumbar vertebra (see Table 1 and Figure 1). Vertebral height has increased from medieval to modern day from 27.6±1.8 mm to 28.7±1.6 mm (p<0.01) in male sample and from 26.2±1.6 mm to 27±1.5 mm (p<0.05) in female sample. Interestingly the vertebral width has decreased from 52.1±3.0 mm to 45.9±3.4 mm (p<0.001) in male sample and from 47.7±3.0 mm to 41.6±2.7 mm (p<0.001) in female sample. These changes are noteworthy for the biomechanical properties of the vertebrae.

**Table 1:** Vertebral dimensions in the study populations.

Sample (sex)	L4height (mm)	L4width (mm)	L4AP (mm)	L4CSA (cm^2^)
Modern ♂ n = 60	30.5±1.67	48.8±3.66	37.7±3.33	14.5±2.19
Modern ♀ n = 31	28.7±1.59	44.3±2.88	34.1±2.19	11.9±1.47
Blackgate ♂ n = 20	28.1±1.38	52.1±2.93***	37.6±2.62	15.4±1.77
Blackgate ♀ n = 20	26.1±1.75	48.2±3.48***	35.8±3.53	13.6±2.22
Westerhus ♂ n = 20	27.2±2.00**	52.1±3.12***	38.1±3.70	15.6±2.29
Westerhus ♀ n = 32	26.2±1.55*	47.4±2.74***	33.8±2.29	12.6±1.34

Vertebral dimensions in the study populations. L4AP = anteroposterior measurement of L4, L4CSA = vertebral cross sectional area of L4 where CSA = π•a•b, in this case a =  vertebral width/2 and b =  vertebral depth/2. Independent samples test, t-test for equality of means (medieval and modern samples compared) * = p<0.05, ** = p<0.01, *** = p<0.001.

Differences in observed vertebral height between modern and medieval study populations can be explained by differences in trunk height and/or stature. Increase in the mean stature from observed medieval ♂171.0 cm and ♀158.2 cm to modern ♂182.7 cm and ♀165.5 cm [24] would explain the chronological difference in vertebral height as changes in vertebral height and stature are positively correlated in study populations (R = .696).

Both medieval samples are very similar with each other in vertebral dimensions. The difference between sexes in measurements is similar in modern and medieval samples (Table 1). Stature was clearly correlated to vertebral CSA (r = 0.80) and there is also clear correlation between estimated body weight and CSA (r = 0.86) indicating that vertebral CSA is a relatively good size estimator for skeletal specimens. Age related differences in vertebral dimensions were not found.

## Discussion

The temporal increase in vertebral height demonstrated in this study, is possibly associated with stature increase from medieval times. The increase in stature should also lead to an increase in vertebral CSA as there is strong correlation between stature and vertebral size [16], [22], [23]. However the mediolateral dimension of L4 has decreased by over 7% from medieval times to the present day. In fact modern males and medieval females are close to each other in this dimension (modern ♂ 45.9±3.4 mm, medieval ♀ 47.7±3.0 mm) whereas the body weight diffrence between these two samples is significant.

The clear difference in vertebral height between medieval and modern samples can be connected to stature increase, but difference in vertebral width and CSA evoke several questions. Which factors lie behind decreased vertebral width? Was there more physical activity during childhood in medieval times causing more robust skeletal structure or maybe substantial periosteal apposition during adulthood? The bone mineral density of lumbar spine is mostly (∼80% of variance) explained by genetic factors [25]. In light of this example and studies about bone mass heritability [26], [27] also the genetic influences on vertebral strength and especially CSA, could be intense. If there has been genetic relaxation considering vertebral width, has there been enough time for gene pool changes?

Higher levels of physical activity occurring during early life could cause differences in vertebral CSA between the medieval and modern populations due to phenotypic plasticity. The differences in CSA could also be caused by differences in selection mechanisms favoring more robust bone structure during medieval times. A physically more demanding lifestyle could have led to selection for less fragile bones as skeletal fractures would have been severely debilitating or even a cause of death. To bring some light to these questions, some brief experiments were put into practice.

The femur has similar function related to carrying upper body mass as does the lumbar vertebra. Femoral measurements are popular when body mass is estimated for skeletal specimens [28]–[30]. Femur head size reflects the body mass that person was “designed” to support. In other words, femur head size is strongly controlled by genetic factors and it can not be reshaped by the influence of physical activity during adulthood [31]. Another femoral measurement, femoral mid-shaft cross section, is phenotypically flexible in response to physical activity through periosteal- and endosteal remodeling and the cross sectional dimensions of the femur midshaft have high correlations with the person's physical activity level [32].

The dimensions of the 4th lumbar vertebra were compared to femur head diameter and femoral shaft cross section in the Westerhus sample. Both of these measurements had relatively high correlation (femur head R = 0.78, femoral shaft R = 0.79) with the mediolateral width of the vertebra. According to this minor test, vertebral dimensions are connected to both the individual's overall skeletal robusticity and their physical activity level.

In conclusion, vertebral strength has evidently decreased from medieval times but there is no single explanation for the reduction and further studies with more diverse study populations will be needed to resolve the issue. It is well known that overall skeletal robusticity has decreased over time especially alongside technological development [33] very likely affecting the vertebral dimensions as well. Improved healthcare has had an influence on selection mechanisms, and changes in nutrition and physical activity have affected the appearance of the phenotype. Irrespective of the reason(s) for the modern, slender phenotype in the lumbar vertebrae, the differences in biomechanical properties between modern and medieval samples are indisputable.

## Materials and Methods

Measurements for this study were obtained from a sample of modern Finns (n = 91) and two medieval osteoarchaeological samples, Swedish (n = 52) and British (n = 40). The total number of measured individuals was 183 and they were all adults as assessed from medical record in modern samples and by dental development and epiphyseal fusion in medieval samples. Samples have some variation in their geographical origin; however the modern Finns and modern Swedes are genetically quite similar [34]. Both modern populations are large-sized [35–58] thus possessing larger mean skeletal measurements than most of the recent human populations.

The male subjects of the modern study population consisted of 60 Caucasian male paper mill and chemical factory workers (age range 42–54 years) who were randomly selected from a group of 228 volunteers attending as an occupational cohort in a cross-sectional study of the lumbar intervertebral disc. The study was approved by the Oulu University Hospital Ethics Committee. All study members gave written informed consent prior to enrollment and received no compensation. Additionally 31 Caucasian females (age 21–80) were randomly selected from 1194 clinical lumbar spine MRI examinations performed during 2007 at the Oulu University Hospital.

The Swedish Medieval sample consisted of 52 individuals, 32 female and 20 male originating from the early 12th - late 13th century Westerhus. This skeletal collection is well documented [39] and the information about age, sex and also some skeletal dimensions were obtained from the literature. The British sample is referred to as the Newcastle-Blackgate and it consists of 9th–11th century skeletons from the Black Gate Cemetery, Newcastle-upon-Tyne. For this study, the L4 vertebrae of 20 adult male and 20 female adults were examined. Age and sex information for the Blackgate material was obtained from records held at the University of Sheffield [40].

**Figure 2:**
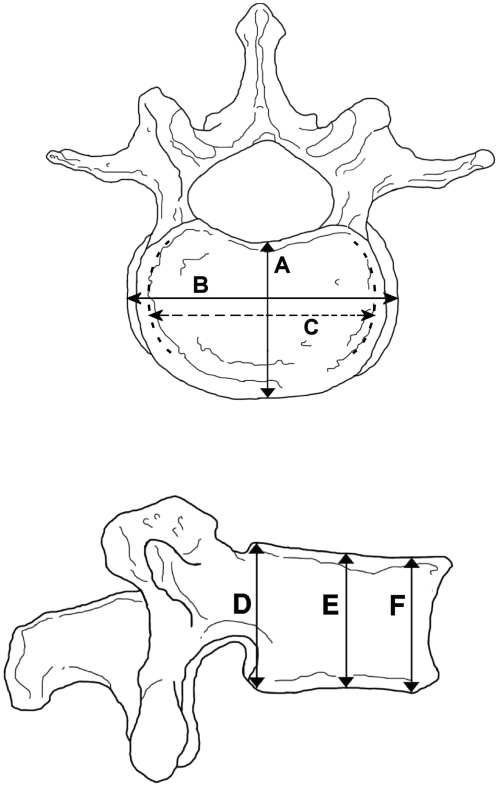
The site of the measurements in L4 vertebra. A: anteroposterior dimension; B: Maximum mediolateral width; C: Minimum mediolateral width; D: posterior height; E. medial height; F: anterior height. Vertebral dimensions in the study populations. L4AP = anteroposterior measurement of L4, L4CSA = vertebral cross sectional area of L4 where CSA = π•a•b, in this case a =  vertebral width/2 and b =  vertebral depth/2. Independent samples test, t-test for equality of means (medieval and modern samples compared) * = p<0.05, ** = p<0.01, *** = p<0.001.

Caliper measurements taken on each vertebra were L4 anterior height, L4 posterior height, L4 maximum mediolateral width, L4 minimum mediolateral width and L4 maximum anteroposterior depth (Figure. 2). All the measurements were taken from vertebral corpus only and were recorded to the nearest 0.1 mm. In addition to individual dimensions and dimension combinations also vertebral cross sectional area (CSA) was used for this study. Vertebral CSA was calculated with following formula (ellipse area) A = π•a•b. In this case a =  vertebral width/2 and b =  vertebral depth/2. This formula has been demonstrated to be accurate for estimating vertebral CSA [23], [41].

Different measuring techniques were required for the modern clinical samples. Modern data were measured from magnetic resonance (MR) images whereas the medieval data were obtained using standard digital measuring calipers. To test the difference between the MRI and measuring caliper techniques, 12 archaeological and cadaver vertebra were soaked in physiological saline solution. The caliper measurements were performed on both wet and dry vertebrae while MRI was performed only on the wet samples. The measurements taken from the “wet” vertebrae were found to be similar regardless of the measuring technique (R = .985). The equation relating the MRI measurement to the dry vertebrae dimensions was determined. The correction factor (MRI measurement = 1.012× dry dimension) was developed by using linear regression to convert the dry bone measurements relative to the MRI measurements. Also the Bland-Altman's test was performed to test the difference between measuring techniques.

The MR imaging was performed using two GE Signa 1.5T scanners (Milwaukee, WI, USA) with a GE torso array coil. Two routine lumbar spine MRI sequences were used for measuring vertebral dimensions. For measuring vertebral height dimensions (anterior, medial and posterior), a T2-weighted fast spin echo sequence (TR = 3000–4000 ms; TE = 117 ms; in-plane resolution of 0.63–0.66 mm in anteroposterior direction and 1.06–1.25 in the superoinferior direction; four averages; 4-mm slice thickness with intersection gap of 0.8–1.0 mm; echo train length of 19–28; 41.7 kHz bandwidth) in the sagittal plane was used. For measuring anteroposterior and mediolateral (maximum and minimum) dimensions, a T2-weighted fast spin echo sequence (TR = 3100–5160 ms; TE = 103–107 ms; in-plane resolution of 0.70–0.78 mm in the left-right-direction and 0.70–1.13 mm in the anteroposterior direction; four averages; 4-mm slice thickness with intersection gap of 0.8–1.0 mm; echo train length of 12–26; 31.2 kHz bandwidth) in the axial plane was used.

Since dry bone samples are known to shrink [42], all of the archeological measurements were transformed relative to MRI measurements to equate vertebra in vivo using the developed correction factor. Both transformed and untransformed values were tested for this study. The analyses represented here are however performed using original, “dry” measurements for archaeological vertebra to avoid potential effects of the transformation process. The archaeological measurements would increase after correction to wet bone values.

Besides temporal trends, the role of age, stature and weight were investigated for vertebral size. Age information was accurate for the modern sample as date of birth was included in the scanning report. For the archaeological samples age and sex estimations were obtained from the literature [39], [40]. Stature estimations were performed for the Swedish medieval sample following Raxter et al. [43] equation 1 (age = 20, prediction error coefficient +0.31 for male and −0.14 for female). Furthermore weight estimations (according to Ruff et al. 2005 [38]) were performed for individuals with stature information.

The geometrical parameters of vertebrae were statistically compared between different samples using the T-test for two independent samples (SPSS 14.0). The correlations between vertebral dimensions with age, stature, body mass and other skeletal dimensions were examined in search of interpretative factors for vertebral size. The results are expressed as mean±SD.

## References

[1] Mays S (1996). Age-dependent cortical bone loss in a Medieval population.. International Journal ofOsteoarchaeology.

[2] Mays S (1999). Osteoporosis in earlier human populations.. Journal of Clinival Densitometry.

[3] Brickley M (2002). An investigation of historical and archaeological evidence for age-related bone loss and osteoporosis.. International Journal of Osteoarchaeology.

[4] Sievanen H, Jozsa L, Pap I, Jarvinen M, Jarvinen (2007). Fragile external phenotype of modern human proximal femur in comparison with medieval bone.. J Bone Miner Res.

[5] Black DM, Palermo L, Nevitt MC, Genant HK, Christensen L (1999). Defining incident vertebral deformity: a prospective comparison of several approaches. The Study of Osteoporotic Fractures Research Group.. J Bone Miner Res.

[6] Melton LJ, Kan SH, Frye MA, Wahner HW, O'Fallon (1989). Epidemiology of vertebral fractures in women.. Am J Epidemiol.

[7] Melton LJ (1997). The prevalence of osteoporosis.. J Bone Miner Res.

[8] Kenny A, Taxel P (2000). Osteoporosis in older men.. Clin Cornerstone.

[9] Odvina CV, Wergedal JE, Libanati CR, Schulz EE, Baylink DJ (1988). Relationship between trabecular vertebral body density and fractures: a quantitative definition of spinal osteoporosis.. Metabolism.

[10] Biggeman M, Hilweg D, Brinckmann P (1988). Prediction of the compressiev strength of vertebral bodies of the lumbar spine by quantative computed tomography.. Skeletal Radiology.

[11] Gilsanz V, Boechat MI, Gilsanz R, Loro ML, Roe TF (1994). Gender differences in vertebral sizes in adults: biomechanical implications.. Radiology.

[12] Vega E, Ghiringhelli G, Mautalen C, Rey VG, Scaglia H (1998). Bone mineral density and bone size in men with primary osteoporosis and vertebral fractures.. Calcif Tissue Int.

[13] Seeman E (1999). The structural basis of bone fragility in men.. Bone.

[14] Ruyssen-Witrand A, Gossec L, Kolta S, Dougados M, Roux C (2007). Vertebral dimensions as risk factor of vertebral fracture in osteoporotic patients: a systematic literature review.. Osteoporos Int.

[15] Gilsanz V, Boechat MI, Roe TF, Loro ML, Sayre JW (1994). Gender differences in vertebral body sizes in children and adolescents.. Radiology.

[16] Duan Y, Turner CH, Kim BT, Seeman E (2001). Sexual dimorphism in vertebral fragility is more the result of gender differences in age-related bone gain than bone loss.. J Bone Miner Res.

[17] Gilliam T, Freedson P, Geenen D, Shahraray B (2008). Physical activity patterns determined by heart rate monitoring in 6–7 year-old children.. Medicine & Science in Sports & Exercise.

[18] Taylor JR, Twomey LT, Corker M (1990). Bone and soft tissue injuries in post-mortem lumbar spines.. Paraplegia.

[19] Seeman E (2002). Pathogenesis of bone fragility in women and men.. Lancet.

[20] Mosekilde L (1989). Sex differences in age-related loss of vertebral trabecular bone mass and structure–biomechanical consequences.. Bone.

[21] Thomsen JS, Ebbesen EN, Mosekilde L (2002). Predicting human vertebral bone strength by vertebral static histomorphometry.. Bone.

[22] Ebbesen J, Thomsen JS, Beck-Nielsen H, Nepper-Rasmussen H (1999). Lumbar vertebral body compressive strength evaluated by dual-energy X-ray absorptiometry, quantitative computed tomography, and ashing.. Bone.

[23] Duan Y, Seeman E, Turner CH (2001). The biomechanical basis of vertebral body fragility in men and women.. J Bone Miner Res.

[24] Niskanen M, Junno J-A (2004). Paleoliittisen kauden eurooppalaisten ruumiin koko.. Muinaistutkija.

[25] Videman T, Levalahti E, Battie MC, Simonen R, Vanninen E (2007). Heritability of BMD of femoral neck and lumbar spine: a multivariate twin study of Finnish men.. J Bone Miner Res.

[26] Pocock NA, Eisman JA, Hopper JL, Yeates MG, Sambrook PN (1987). Genetic determinants of bone mass in adults. A twin study.. J Clin Invest.

[27] Arden NK, Spector TD (1997). Genetic influences on muscle strength, lean body mass, and bone mineral density: a twin study.. J Bone Miner Res.

[28] McHenry H (1988). New estimates of body weight in early hominids and their significance to encephalization and megadontia in 〈〈robust〉〉 australopithecines.. Evolutionary History of the 〈〈Robust〉〉 Australopithecines.

[29] McHenry H (1992). Body size and proportions in early hominids.. American Journal of Physical Anthropology.

[30] Ruff C (2000). Prediction of body mass from skeletal frame size in elite athletes.. American Journal of Physical Anthropology.

[31] Ruff C (2002). Variation in human body size and shape.. Annual Review of Anthropology.

[32] Ruff C (2008). Femoral/humeral strength in early African *Homo erectus*.. Journal of Human Evolution.

[33] Ruff C (2006). Gracilization of the modern human skeleton - the latent strength in our slender bones teaches lessons about human lives, current and past.. American Scientist.

[34] Niskanen M (2002). The origin of the Baltic-Finns from the physical anthropological point of view.. Mankind Quarterly.

[35] Lundberg H, Linders F (1926). The Racial Characters of the Swedish Nation.. Anthropologica Suecica.

[36] Kajanoja (1971). A Study in the Morphology of the Finns and its Relation to the Settlement of Finland.. Annales Academiae Scientiarum Fennicae.

[37] Dahlström S (1981). Body build and physique of young Finnish adults: studies based on inductee surveys and anthropometric measurements on 223 conscripts..

[38] Ruff C, Niskanen M, Junno J-A, Jamison P (2005). Body mass prediction from stature and bi-iliac breadth in two high latitude populations, with application to earlier higher latitude humans.. Journal of Human Evolution.

[39] Gejvall N-G (1960). Westerhus..

[40] A.R.C.U.S. (1996).

[41] Peel N, Eastell R (1994). Diagnostic value of estimated volumetric bone mineral density of the lumbar spine in osteoporosis.. Journal of Bone and Mineral Research.

[42] Lanier R (1939). The Presacral Vertebrae of American White and Negro Males.. Am J Phys Anthropol.

[43] Raxter MH, Auerbach BM, Ruff C (2006). A revision of the Fully technique for estimating statures.. American Journal of Physical Anthropology.

